# Awareness and utilization of modern contraceptives among street women in North-West Ethiopia

**DOI:** 10.1186/1472-6874-12-31

**Published:** 2012-10-02

**Authors:** Berihun Megabiaw

**Affiliations:** 1 Department of Epidemiology and Biostatistics, Institute of Public Health, College of Medicine and Health Sciences, University of Gondar, P.O. Box 1288, Gondar, Ethiopia

## Abstract

**Background:**

Contraception is a major component of reproductive health. Assessing the levels of contraceptive awareness and use helps to identify potential areas of intervention. Hence, this study was conducted to assess awareness, practice and associated factors of modern contraceptives among street women in North-West Ethiopia.

**Methods:**

A cross-sectional study was conducted on 204 street women from Gondar and Bahir Dar cities. Participants were recruited from “cluster” sites such as main road sides, isolated slum areas, around Churches and/or Mosques (in the mornings of Sundays and other religious feast days) and streets where street women usually reside and/or sleep. Data were collected using a pre-tested and structured interview questionnaire in local language (Amharic) after informed verbal consent. Data were then entered into SPSS version 16.0 for analysis. Binary logistic regression models were fit to assess associations and control confounding. Associations were measured by the Odds ratio and its 95% confidence interval.

**Results:**

The mean (±SD) age of participants was 30.9 (± 8.7) years. Majority (90.7%) had ever heard about modern contraceptives. Nearly half (47.1%) had ever used and a third (34.3%) were current users. Three quarter of the current users (74.3%) were using injectables while 10% were on long acting or permanent methods. Marital status (AOR=2.81), family size (AOR=2.67) and age of 25–34 years (AOR=3.45) were associated with modern contraceptive use.

**Conclusions:**

Current contraceptive use among street women is satisfactory considering their life styles and living conditions. However, further research is required to explain perceptions and hidden barriers.

## Background

The increasing growth of population has become an urgent problem in Ethiopia. The Ethiopian population grew at an alarming rate from about 40 million in 1984 to 54 million in 1994 and about 74 million in 2007 [1]. Currently, Ethiopian population is growing more than double the average global population growth rate (2.60% Vs 1.13%) [1,2]. Ethiopia has a high maternal mortality rate (676 per 100,000 live births) [3]. Contraceptive use can improve maternal health and is one of the strategies to achieve improved maternal health worldwide [4].

Poor reproductive health causes widespread hardship to families and communities, particularly in the developing world [5], where maternal mortality is high. Actions to address these problems include the provision of quality family planning services, emergency obstetric care, post-abortion care, and prevention and treatment services for HIV/AIDS and other sexually transmitted infections [5]. Family planning is one of the highly advocated options in developing countries to control the fast population growth and to decrease the higher maternal and child deaths though it has steadily decreased as an international priority in recent years [6,7].

People in developing countries, poorer ones, and the less educated are more likely to have many children [6]. It is immensely important that women at any physical, social or economical status decide on their family planning choices. Their decision and utilization mainly depends on the contraceptive knowledge and access for family planning options [8] and the couple’s educational status [9]. In Ethiopia, educated women and those in the highest wealth quintile are most likely to use contraception [10].

Women living in the streets are less likely to benefit from basic reproductive health services as living in the poorest segment, delivering and caring their children out of streets life. As street people, girls and women in particular, are exposed to sexual exploitation, rape and prostitution, it is important that efforts are done to let them use contraceptive options to prevent unwanted pregnancy and sexually transmitted diseases along with the prevention of sexual harassment.

Several studies had been done on the family planning prevalence (usually modern contraceptives) among women [11-14] though none addressed contraceptive awareness and utilization among street women who are economically, socially and nutritionally disadvantaged. As part of the population, street women have the right to family planning information and access. It is a frequent observation to see women begging on the streets of major cities in Ethiopia having one or two babies by their sides. However, data about contraceptives awareness and utilization is scarce among street women. Hence, this study aimed to assess levels of awareness, utilization and associated factors of street women in North-West Ethiopia.

## Methods

### Study design

A cross-sectional study was conducted in Gondar and Bahir Dar cities of North-West Ethiopia in October 2010.

### Study area

The study was conducted in two major cities (Gondar and Bahi Dar) of Amhara National Regional State. These two cities are among the major cities in the region in which a significant proportion of the population of the region lives. Bahir Dar and Gondar cities, located at 550 and 727 km from Addis Ababa (the capital city of Ethiopia) respectively. Having a population of about 182,676 and 206,987 respectively [1] these two cities are growing fast. Bahir Dar is the capital city of Amhara National Regional State and Gondar is the capital city of North Gondar Administrative Zone. According to the 2006 annual report of Amhara National Regional State Bureau of Labor and Social Affairs, of the 3576 street people in 32 urban areas of the region 43% were from these two cities [15]. In both cities, there were houses built by the government for beggar people with families.

### Sampling

After selecting “cluster” sites in both cities, a total of 204 street women of childbearing age living in the streets, verandas or compounds of churches and mosques during the data collection period who were begging, sleeping at night by road sides and residing in plastic houses at isolated areas and camps were interviewed.

Women who make their lives on street life by begging, sleep at streets or road sides were defined as *street women*. Those who had no formal homes (homeless) and sleep on streets, verandas, balconies, etc. at night were classified as “on street” while those who have houses to go for sleep at night while making their lives on street life were termed as “off street”.

### Data collection

Data collected from street women in the morning during Sundays and other religious feast days at main roads to Churches and Mosques (where street people are frequently observed begging sitting in queues) and interviewed them individually. At night time (after 9:30 PM) street women were interviewed at streets and road sides where homeless women reside. Data collectors were five final year health officer students trained for this purpose. The contents of the interview questionnaire were structured into three sections (socio-demography, knowledge and practice questions about modern contraceptives). Pre-tested was done on 20 women (10 from each city) who were excluded from further analysis.

Women’s awareness towards modern contraceptives was assessed using simple knowledge questions about contraceptives (if they have ever heard, able to mention methods etc.) and women were said to be practicing contraception if they were using one of the modern contraceptive methods to prevent unwanted pregnancy or to space births.

### Data analysis

The data were entered into a computer using Epi Info 2002 (CDC, Atlanta, Georgia) and exported to SPSS Version 16.0 (SPSS Inc, Chicago). Data were analyzed using descriptive statistics and logistic regression (binary and multiple) analyses to determine the effect of factor(s) on the outcome variable(s) and to control possible confounders. *P-value* < 0.05 was considered to show statistical significance. Factors found to have a p-value ≤ 0.2 in the binary logistic regression were further entered into the multivariate analysis. Owing to some missing answers to certain survey questions, the denominator used in percentage computation varies according to the responses obtained for each survey question.

Ethical approval was obtained from the Research and Publications Office (RPO) of the College of Medicine and Health Sciences, University of Gondar. Permission letters to conduct the research were obtained from both city administration labor and social affairs offices. The purpose of the study was explained to each participant and verbal consent was obtained regarding agreement to participate in the study after the information and consent forms were read to them in Amharic language. As many of them were not able to read and write, written consent was not possible. No names were recorded to keep the identity of respondents anonymous. After completing data collection, health education was given for people living on streets about the benefits and on the how and where to gets of modern contraceptives. No incentives were given for individual women.

## Results

Of the 204 women included in the study, 113(55.4%) from Gondar and 91(44.6%) were from Bahir Dar. The mean age of participants was 30.9 ± 8.7 SD years. Nearly two third (65.2%) were rural residents before they got engaged in street life. Sixty three (30.9%) were married. Majority (88.2%) were Orthodox Christians and Amhara ethnics (96.1%) (Table 1).

**Table 1:** Socio-demographic characteristics of street women, Northwest Ethiopia (n=204)

**Variables**	**Number (%)**
**Address**	
Gondar	113 (55.4)
Bahir Dar	91 (44.6)
**Age (years)**	
15–24	53 (26.0)
25–34	71 (34.8)
35-49	80 (39.2)
**Residence before street life**	
Urban	71 (34.8)
Rural	133 (65.2)
**Religion**	
Orthodox	181 (88.7)
Muslim	23 (11.3)
**Marital status**	
Single	56 (27.5)
Married	63 (30.9)
Divorced	43 (21.1)
Widowed	36 (17.6)
Separated	6 (2.9)
**Educational Status**	
Cannot read & write	139 (68.1)
Read & write only	30 (14.7)
Elementary (grade 1–8)	35 (17.2)
**Disability**	
Yes	83 (40.7)
No	121 (59.3)
**Type of disability**	
Blindness	22 (26.5)
Problem on hand or leg	20 (24.1)
Non specified internal problem	27 (32.5)
Mental health problem	10 (12.0)
Difficulty hearing	2 (2.4)
Other	2 (2.4)
**Have dependent family member**	
Yes	107 (52.5)
No	97 (47.5)
**Sleeping at night**	
“On street”	61 (29.9)
“Off street”	143 (70.1)

Ninety five (46.6%) were sexually active in the six months prior to data collection. Nearly half (48.0%) had history of pregnancy, of which 44 (21.6%) had more than one pregnancy after engaged in street life. More than a quarter (26.5%) reported that they had history of sexual assault while living in streets.

**Figure 1:**
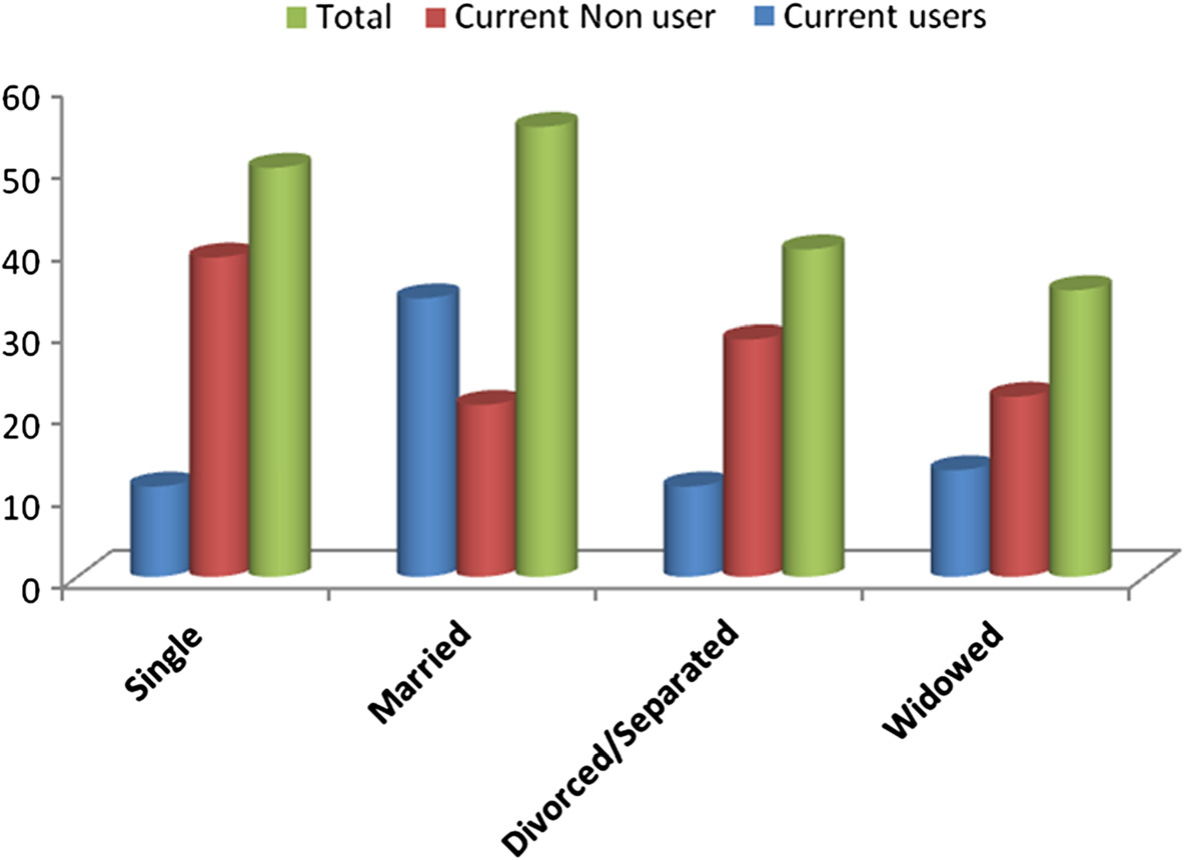
Modern Contraceptive use among street women by marital status, NW Ethiopia, October 2010.

Hundred eight five (90.7%) said they had heard about modern contraceptives and 179 (87.7%) agreed that it is possible to control birth. Almost half (47.1%) of the women in this study had ever used one of the modern contraceptives while 70 (34.3%) were currently using modern contraceptives (Table 2). Almost three quarters (74.3%) of the current users use injectable contraceptive methods followed by oral contraceptive pills (11.4%). More than half (54.0%) of married women were currently using modern contraceptives (Figure 1).

**Table 2:** Awareness and utilization of street women towards modern contraceptive methods, Northwest Ethiopia (n=204)

**Variable**	**Number (%)**
**Births can be controlled**	
Yes	179 (87.7)
No	25 (12.3)
Methods mentioned for birth control *	
Modern contraceptives	163 (79.9)
Condom	43 (21.1)
Safe period	13 (6.9)
**Has information about modern contraceptives**	
Yes	190 (93.1)
No	14 (6.9)
Source of information about modern contraceptives*	
Radio and TV	89 (43.7)
Health facility/Education	77 (37.7)
Neighbors	71 (34.8)
Friends/Relatives	67 (32.8)
Voluntary clubs	21 (10.3)
Print materials	3 (1.5)
Modern contraceptives known by respondents*	
Injectable	172 (84.3)
Pills	138 (67.6)
Norplant	67 (32.8)
Condom	59 (28.9)
Tubaligation	24 (11.8)
Other	6 (2.9)
Place to get modern contraceptives*	
Health Centre/Clinic	178 (87.3)
Pharmacy	47 (23.3)
Other (Clubs, Volunteers)	5 (4.0)
Advantages of modern contraceptives mentioned*	
Prevent unwanted pregnancy	134 (65.7)
Spacing births	110 (53.9)
Limit births	88 (43.1)
Induce abortion	30 (14.7)
Other	9 (4.4)
**Ever use of modern contraceptives**	
Yes	96 (47.1)
No	108 (52.9)
**Current modern contraceptive use**	
Yes	70 (34.3)
No	134 (65.7)
**Contraceptives methods currently being used**	
Injectable	52 (74.3)
Pills	8 (11.4)
Norplant	5 (7.1)
Condom	3 (4.3)
Other (Tubaligation & IUCD)	2 (2.9)
**Future plan to use modern contraceptives**	
Yes	62 (46.3)
No	72 (53.7)

*Total may be greater than 100% as there are multiple responses, *IUCD* Intrauterine Contraceptive Device.

Of the 134 women who were not currently using any of the modern contraceptive methods, 62 (44.9%) need to use them in the future. The most frequent reason for not using contraceptives was fear of side effects (35.9%) followed by being sexually not active (27.4%).

### Factors associated with modern contraceptive use

Logistic regression analysis was conducted to assess possible relationship between current modern contraceptive use and other explanatory variables (Table 3). From all variables considered and entered in the multivariable analysis, being married, aged between 25–34 years, having at least one alive child, histories of pregnancy and/or sexual assault while at street life were found to be significantly associated with current modern contraceptive use.

**Table 3:** Logistic regression of associated factors with current contraceptive use among street women in Northwest Ethiopia

**Predictor variable**		**Contraceptive use**			**COR(95%CI)**	**AOR(95% CI)**	**P-value**
		**Yes**	**No**	**Total**			
Age	15-24	15	38	53	1.11(.51,2.41)	1.31(.55,3.10)	0.18
	25-34	34	37	71	2.58(1.31,5.11)*	2.81(1.39,5.67)*	0.004
	35-49	21	59	80	1.00	1.00	
Previous Residence	Urban	24	48	72	.94(.51,1.72)	1.26(.64,2.47)	0.50
	Rural	46	86	132	1.00	1.00	
Marital Status(current)	Unmarried	36	105	141	1.00	1.00	
	Married	34	29	63	3.42(1.75,6.70)*	3.45(1.81, 6.97)*	0.001
Family size	Alone	8	30	38	1.00	1.00	
	2-3	29	54	83	2.01(.80,4.96)	2.05(.78,5.67)	0.144
	>4	33	50	83	2.48(1.01,6.06)*	2.67(1.03,6.93)*	0.044
Have at least one alive child	Yes	56	89	145	2.02(1.02,4.02)*	2.54(.97,6.64)	0.057
	No	14	45	59	1.00		
History of pregnancy at street life	Yes	42	56	100	2.09(1.06,3.76)*	2.0(.92,4.32)	0.079
	No	28	78	106	1.00	1.00	
Sexual assault at street life	Yes	27	43	70	2.49(1.31,4.72)*	2.32(1.05,5.13)*	0.038
	No	27	107	134	1.00	1.00	

* Significant at p-value <0.05.

Street women aged 25–34 years old were found to be about 3 times (AOR=2.81 & 95%CI:1.39,5.67) highly likely to use modern contraceptives compared to those aged 35 years or older. Likewise, married women were 3.5 times (AOR=3.45 &95%CI:1.81,6.97) more likely to be current modern contraceptive users compared to currently unmarried women (single, divorced, widowed or separated). Women who have at least one child alive had a two and half times higher odds of using modern contraceptives as compared to women without an alive child though it was not significant when adjusted for potential confounders. Women having a higher family size (≥5) were two and half times (AOR=2.67&95%CI:1.03,6.93) highly likely to be currently using modern contraceptives compared to their counterparts.

## Discussion

The current study explored contraceptive issues on a section of the population that is largely ignored. The fact that majority (93.1%) of the street women had ever heard about modern contraceptives in this group of the population is of paramount importance. This result is in line with the 2011 Ethiopian Demographic Health Survey report which reported that knowledge of contraception was nearly universal in Ethiopia [3]. However, it is higher than similar reports among married urban dwellers [10-12,16]. The difference may be partly explained by the time gap between studies and partly by the wide scale increase of information and access to contraceptives in Ethiopia [3].

Similar to other studies around Gondar [11-13], injectables were largely known by respondents (84.3%) followed by Pills (67.6%), Norplant (32.8%) and condom (28.9%). This shows that modern contraceptives were well known and popular among this group of women. The possible reason for this can be that, being in urban areas, these groups of women are usually addressed by health education via media which emphasis on the dual effect of condom as part of sexually transmitted infection prevention efforts. Additionally, the study areas were two major cities in the region where there are various governmental and non-governmental organizations working on various angles of maternal health issues.

Ninety six women (47.1%) had ever used modern contraceptives. About a third (34.3% were currently using modern contraceptives. It’s well known that current use is a better measure of contraceptive prevalence as it measures the actual contraceptive practice at a particular point in time and correctly determines the reduction of fertility attributable to the contraceptive [17]. This current contraceptive usage is comparable to other studies conducted in urban areas of Ethiopia which reported a 22.8-35.5% current contraceptive use [12,14]. This can be attributed to the increasing family planning information and service nationwide [3]. In this study, current contraceptive use is higher among currently married street women (54.0%) than the unmarried counterparts (21.8%) which agrees with the fact that marital status is a universal predictor of contraceptive use [16].

The wide use of injectable contraceptive methods (74.3%) is in agreement with several studies in Ethiopia and other African countries [11-13,17] which might have resulted from the wider availability. The use of condom as a family planning method in this group of women (5 women were using condom and another method) is encouraging and of advantage for its dual prevention. This result also showed that 10% of the methods being used were long acting or permanent methods (5 on Norplant, 1 on Intrauterine device and 1 Tubaligation) which indicates their need to space for longer periods or limit births at all among street women.

The results showed that apart from the basic predictor- marital status of the woman, women’s age ranging from 25–34 years, having at least one alive child, being sexually active in the last six months, history of pregnancy and/or sexual assault while on street life were the factors associated with current contraceptives use. This may be due to the perceived higher risk of unwanted pregnancy they face and the higher magnitude of sexual assault (26.5%) among street women. The association between age of 25–34 years with contraceptive use was similar to other studies conducted elsewhere [11,14,16]. It can be partly explained by the fact that this age group is usually the age a woman is more likely married and have a child or more in a country such as Ethiopia where the median ages at first marriage and delivery are 16.1 and 19.0 years respectively [10]. In Ethiopia, a woman aged 25–34 years has, at an average, 2–5 children [10] and from this result if a woman has a child then she is likely to use modern contraceptives.

Contraceptive use is often influenced by such characteristics as education, place of residence and wealth where the poor are the most disadvantaged and less likely to use contraceptives [18]. There was no difference between those who came from urban and rural areas with regard to their contraceptive use. This may be explained by the fact that people in the streets, despite their origin were similar once engaged in street life. Though it is universal that urban dwellers have better access to family planning services, and they are more likely to be better educated, street women though considered as urban dwellers were largely not educated. This study was not intended to see effect of wealth differences on contraceptive use as it only assessed the poorest segment of women alone.

This study was conducted only in two cities of the region, which makes generalization to wider areas difficult as the nature of street dwelling made it difficult to obtain a representative sample. It also shares limitations of cross-sectional study designs.

## Conclusions

The current contraceptive use among street women in Gondar and Bahir Dar cities is satisfactory considering the natures of their life styles and living conditions. Married women, women having at least one alive child, those aged 25–34 years were most likely to use modern contraceptive methods. Fear of side effects was mentioned by a large number of women as a reason for not using modern contraceptives.

Since static family planning services are not accessible to street people, other strategies like outreach methods should be considered. Further research is required to explain perceptions and barriers towards modern contraceptive use among this group of women.

## Competing interests

The author declares that he has no competing interests.

## Authors’ contributions

BM is the sole author of this paper.

## Pre-publication history

The pre-publication history for this paper can be accessed here:

http://www.biomedcentral.com/1472-6874/12/31/prepub
